# Changes in CYP3A4 Enzyme Expression and Biochemical Markers Under Acute Hypoxia Affect the Pharmacokinetics of Sildenafil

**DOI:** 10.3389/fphys.2022.755769

**Published:** 2022-01-27

**Authors:** Juanhong Zhang, Rong Wang

**Affiliations:** ^1^College of Life Science, Northwest Normal University, Lanzhou, China; ^2^Key Laboratory for Prevention and Remediation of Plateau Environmental Damage, 940th Hospital of Joint Logistics Support Force of CPLA, Lanzhou, China; ^3^School of Pharmacy, State Key Laboratory of Applied Organic Chemistry, College of Chemistry and Chemical Engineering, Lanzhou University, Lanzhou, China

**Keywords:** acute hypoxia, CYP3A4, sildenafil, pharmacokinetics, blood gas, pathological and biochemical indicators

## Abstract

To investigate the effects of pathological, physiological, biochemical and metabolic enzymes CYP3A4 on the pharmacokinetics of sildenafil under acute hypoxia, rats were randomly divided into the plain group (50 m above sea level), acute plateau group 1 (2300 m above sea level), and acute plateau group 2 (4300 m above sea level), and blood samples and liver tissues were collected. Our results showed that the blood gas, physiological and biochemical indexes of rats changed under acute hypoxia, and the protein expression of CYP3A4 enzyme decreased. The process of absorption, distribution, metabolism and excretion of sildenafil in rats has changed. Compared with the P group, the area under the drug-time curve and the average resident in the H2 group increased to 213.32 and 72.34%, respectively. The half-life and peak concentration increased by 44.27 and 133.67%, respectively. The clearance rate and apparent distribution volume decreased to 69.13 and 46.75%, respectively. There were no statistical differences in the pharmacokinetic parameters between the P group and the H1 group. In conclusion, the pharmacokinetic changes of sildenafil have a multi-factor regulation mechanism, and changes in blood gas, pathology, and biochemical indicators and metabolic enzymes affect the absorption, distribution, excretion, and metabolism of sildenafil, respectively. This study provides experimental evidence and new ideas for the rational use of sildenafil under acute hypoxic conditions.

## Graphical Abstract

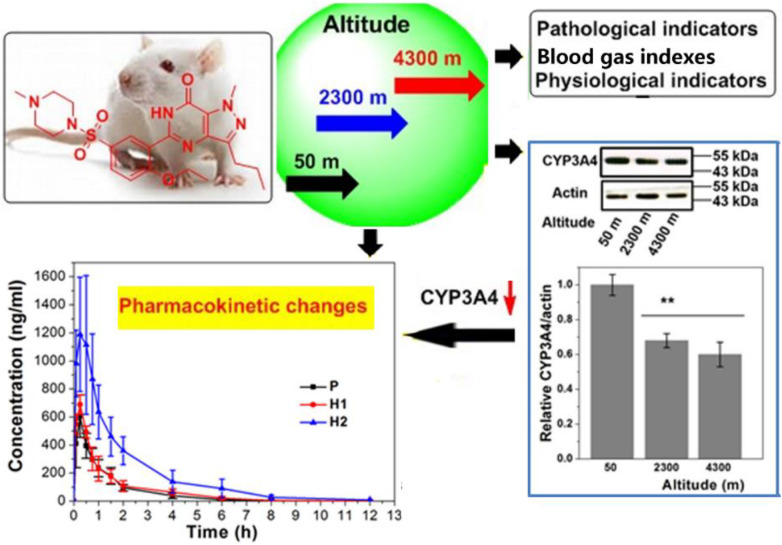

## Highlights

- We first demonstrated that the pharmacokinetic changes of sildenafil have a multi-factor regulation mechanism under acute hypoxia.
- We demonstrated that these changes were mainly caused by the decrease in protein expression of CYP3A4 enzyme under acute hypoxic conditions.
- Our investigation provides experimental evidence and new ideas for the rational use of sildenafil under acute hypoxic conditions.

## Introduction

At present, under high altitude hypoxia conditions, the research on the factors affecting the changes of pharmacokinetic parameters mainly focuses on drug-metabolizing enzymes. The changes in pharmacokinetic characteristics are closely related to the changes in the expression and activity of drug-metabolizing enzymes. The expression and activity of P450 enzymes are affected by hypoxia, which in turn affects the metabolism of related substrates. Previous studies have shown that compared with rats in the plain group, the activity and protein expression of CYP2C9 in the middle-altitude acute hypoxia group and the high-altitude acute hypoxia group have no significant changes, while acute altitude hypoxia may reduce the activity of CYP3A4, which in turn affects the metabolism of its substrate *in vivo* (Jurgens et al., 2002; Duan et al., 2020; Yuan et al., 2020). However, for some enzymes, the degree of influence is inconsistent or controversial, and further experiments are required according to specific conditions. Specifically, under acute hypoxic conditions, the activity of CYP450 enzymes *in vivo* will be affected to varying degrees, further affecting the pharmacokinetic characteristics of the relevant substrates (Ku et al., 2008; Zeng et al., 2017; Sychev et al., 2018), thus studying the expression of CYP450 enzymes and changes in activity are important for the clarification of the metabolic characteristics of the relevant substrate under acute hypoxic conditions. It is a direction worthy of further exploration in the research of rational use of drugs on the plateau.

Sildenafil has been found to have anti-hypoxia effects and is a widely used oral drug (Ku et al., 2008; Bates et al., 2011). It is rapidly absorbed by the gastrointestinal tract after oral administration, and the absolute bioavailability is about 40%. It is mainly cleared by the liver microsomal enzyme CYP3A4, and its second clearance pathway is CYP2C9 (Hyland et al., 2001; Tang et al., 2020). The main N-demethyl metabolite is about half of the parent compound *in vitro*, so about 20% of its pharmacological action may come from N-demethyl metabolites (Hyland et al., 2001; Ku et al., 2008). Changes in drug metabolism and pharmacokinetic properties are important for the pharmacological activity, toxicology, and safety of sildenafil (Francis and Corbin, 2005; Tang et al., 2020; Lee et al., 2021). There are many studies on the pharmacokinetics of sildenafil, but there are no pharmacokinetic data under acute hypoxia (Mekjaruskul and Sripanidkulchai, 2015; Hakamata et al., 2016; Ghoneim and Mansour, 2020; De Bie et al., 2021; Salerno et al., 2021). The change in the metabolic enzymes CYP2C9 and CYP3A4 under acute hypoxic conditions plays an important role in the metabolism of sildenafil, which has aroused great interest in our research. The expression changes and activity changes of CYP2C9 and CYP3A4, which are the main metabolic enzymes of sildenafil (Tang et al., 2020), have a highly important effect on their metabolism under acute hypoxic conditions.

Previous work showed that the expression and activity of the metabolic enzyme CYP2C9 under acute hypoxia were not affected, and the expression of CYP3A4 may be altered (Wang et al., 2017). As part of our continued exploration of high altitude hypoxia-mediated drug metabolism (Zhang et al., 2018, 2019, 2020), the change of CYP3A4 with increasing altitude and the effect of this alteration on the pharmacokinetics of its substrate sildenafil are issues of our concern. Therefore, we report here for the first time that the pharmacokinetic changes of sildenafil under acute hypoxic conditions have a multifactorial regulatory mechanism, and prove that these changes are mainly caused by the decrease of CYP3A4 protein expression under acute hypoxic conditions. Our research provides experimental evidence and new ideas for the rational use of sildenafil under acute hypoxic conditions.

## Materials and Methods

### Chemicals and Reagents

The sildenafil citrate tablets (lot no.1283007) were purchased from Pfizer Pharmaceuticals Ltd (New York NY, United States). The standard substance of sildenafil citrate (lot no. 100783-200401) was obtained from the China Drug and Biologic Product Standardization Station (Beijing, China). HPLC-grade acetonitrile and ammonium formate were purchased from Merck KGaA (Darmstadt, Germany). Other reagents are of analytical grade.

### Apparatus

The Ultra-Fast LC (UFLC, Agilent Technologies, United States), API 3200 MS system (Applied Biosystems, United States), an automatic blood gas system (ABL80, Denmark) and automatic biochemistry analyzer (LX20, United States) were used.

### Animal Groups

Twenty-four Wistar rats (6–8 weeks, weighing 180–220 g, certificate: 2007000524909) were divided into three groups in random as the low altitude group of 50 m in Shanghai (P), the short-term exposure to high altitude group of 2300 m in Xining of Qinghai province (H1) and the short-term exposure to high altitude group of 4300 m in the Huashixia town of Qinghai province (H2). Rats in group H1 were airlifted from Shanghai to Xining by plane and bus 7 h later, involving a distance of about 2250 kilometers. Rats in the H2 group were traveled from Shanghai to Huashixia Town by plane and bus in 15 h, a journey of about 2900 kilometers. Rats in the H1 and H2 groups started relevant experiments 24 h after reaching the destination. The study of the P group was performed at the second military medical university. All experiments were executed according to the guidelines and regulations of the Chinese Association for Laboratory Animal Sciences.

### Measurements of Blood Gas Parameters

The rats were anesthetized by intraperitoneal injection of 10% chloral hydrate, and blood samples were collected from the abdominal aorta for blood gas analysis. The blood gas indicators included blood pH, standard bicarbonate (SBC), buffer base (BB), base access (BE), carbon dioxide partial pressure (PaCO_2_), arterial oxygen partial pressure (PaO_2_), arterial oxygen saturation (SaO_2_), Hemoglobin (Hb), Lactic acid (Lac), sodium ion concentration (cNa^+^), potassium ion concentration (cK^+^) and calcium ion concentration (cCa^2+^) (Wang et al., 2016).

### Pharmacokinetic Study

The treatment methods of the three groups were the same, *i.e.*, after the rats have fasted overnight, the animal dose was converted according to the normal dose taken by the human body, and each rat was given 4.8 mg/kg (aqueous solution) sildenafil by gavage. A series of blood samples (0.2 mL) were collected from the orbital vein at 0.083, 0.25, 0.5, 0.75, 1, 1.5, 2, 4, 6, 8, 12, and 24 h after administration and placed in a heparinized centrifuge tube. The blood sample was then centrifuged at 3000 × *g* for 5 min to obtain plasma, which was stored at −20°C until quantitative analysis.

For analysis, aspirate 30 μL of plasma, add 75 μL of acetonitrile, vortex for 1 min, and then centrifuge at 13,000 × *g* for 5 min. Aspirate the supernatant for liquid chromatography-mass spectrometry (LC/MS/MS) analysis.

### Measurements of Plasma Biochemical Parameters

Blood was collected from the rat orbital venous plexus and centrifuged at 3000 r⋅min^–1^ for 10 min for biochemical analysis. The indexes included alkaline phosphatase (ALP), total protein (TP), albumin (ALB), aspartate aminotransferase (AST), alanine aminotransferase (ALT), total bilirubin (TBIL), direct bilirubin (DBIL), C-reaction protein (CRP), total cholesterol (TCHO), blood urea nitrogen (BUN), creatinine (Cr) and uric acid (UA) (Li et al., 2015; Wang et al., 2016).

### Observation of Liver Histomorphology and Measure of CYP3A4 by Western Blotting

After the rats were anesthetized, the liver was taken out and washed with 0.9% NaCl saline, and the part was then fixed with 10% formaldehyde. The other part uses western blotting to analyze the expression of CYP3A4.

### Data Analysis

Software DAS 2.0 was used to analyze the pharmacokinetic parameters of sildenafil. All data were expressed as mean ± SD. Analysis of statistical significance was performed, and *P* < 0.05 was considered to indicate a statistically significant result. The analysis was carried out using SPSS software, version 13.0.

## Results

### Method Validation

**FIGURE 1:**
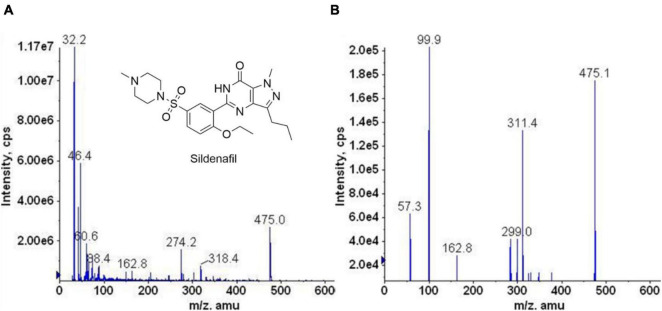
The chemical structure of sildenafil and its MS/MS spectrum. **(A)** Mass-spectrogram of the parent ion. The illustration shows the chemical structure of sildenafil. **(B)** Mass-spectrogram of fragmentation.

The mass spectrum scan of sildenafil is shown in Figure 1, and the detection ion pair is *m/z* 475.0→99.9. Ion spray voltage (IS): 5500 V, ion source temperature (TEM): 250°C, collision energy (CE): 38 eV, dissociation voltage (DP): 50 V. The shim-pack XR-ODS column (3.0 mm × 75 mm, 2.0 μm) chromatographic column is performed. The mobile phase is a mixture of acetonitrile: 2 mM ammonium formate (85:15, v/v).

**FIGURE 2:**
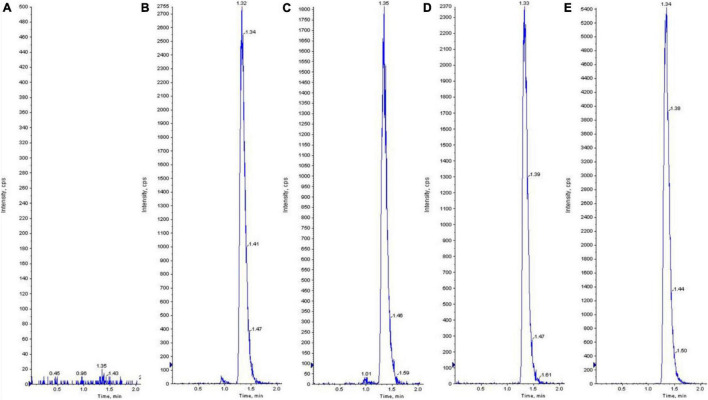
Determination of the typical chromatograms of sildenafil in rat plasma. **(A)** The samples of blank plasma. **(B)** The blank plasma was spiked with standard sildenafil of 100 ng⋅mL^–1^. **(C)** The rat plasma 2 h after intragastric administration of sildenafil at low altitude. **(D)** The rat plasma 2 h after intragastric administration of sildenafil at the high altitude of 2300 m. **(E)** The rat plasma 2 h after intragastric administration of sildenafil at the high altitude of 4300 m.

The chromatogram of sildenafil is shown in Figure 2. The retention time is 1.33 min, and there is no matrix interference nearby, which is suitable for plasma sample analysis. The standard curve equation is *Y* = 187X-25.4, and the correlation coefficient is 0.9993. The linear relationship is good in the range of 2.5∼4000 ng⋅mL^–1^. Plasma samples of sildenafil were placed at room temperature for 24 h, three times of repeated freezing and thawing, and stored at 4°C for 1 month, and then taken out for determination following the law. The RSD% are 1.09, 0.98, 3.80%, respectively, indicating that the sample is stable under the above conditions. The method is sensitive, accurate, and simple, and can be used to study the pharmacokinetics of sildenafil in rats.

### Analysis of Blood Gas Indexes

Changes in physiologic parameters induced by acute hypoxia are shown in Table 1. Compared with the low altitude group, the SBC and SaO_2_ of the acute exposure group (H1) were significantly decreased by 19.04 and 16.72%, respectively, while the Lac was significantly increased by 54.74% (*P* < 0.05). The SBC, PaCO_2_, PaO_2_, and SaO_2_ values of the acute exposure group (H2) were significantly decreased by 3.10, 29.63, and 32.50%, respectively, while the pCO_2_ and pO_2_ were remarkably increased by 15.09 and 17.81%, respectively (*P* < 0.05).

**TABLE 1:** Results of blood gas analysis in rats at different groups (mean ± SD, *n* = 8).

Blood gas indicators	Low altitude (P)	Acute exposure (H1)	Acute exposure (H2)
pH	7.43 ± 0.03	7.37 ± 0.04	7.45 ± 0.02
SBC (mmol/L)	25.63 ± 0.82	21.53 ± 0.25*	22.17 ± 0.51*
BB (mmol/L)	1.58 ± 0.57	−1.85 ± 0.29*	−2.86 ± 1.19*
BE (mmol/L)	1.42 ± 0.73	−2.2 ± 0.52*	−5.30 ± 1.15*
PaCO_2_ (mmHg)	41.83 ± 2.39	40.50 ± 3.67	26.90 ± 1.40*^#^
PaO_2_ (mmHg)	102.13 ± 2.62	87.5 ± 4.59*	55.91 ± 4.77*^#^
SaO_2_ (%)	98.30 ± 0.54	96.33 ± 0.85	90.24 ± 2.08*^#^
Hb (g/dL)	12.99 ± 0.35	13.32 ± 0.87	15.87 ± 1.00*
Lac (mmol/L)	1.37 ± 0.28	2.12 ± 1.04*	2.90 ± 0.95*^#^
cNa^+^(mmol/L)	140.59 ± 1.29	140.86 ± 1.06	145.73 ± 1.74*^#^
cK^+^(mmol/L)	4.19 ± 0.37	4.35 ± 0.53	4.30 ± 0.21
cCa^2+^(mmol/L)	1.19 ± 0.05	1.32 ± 0.05*	1.30 ± 0.04*

*P < 0.05 versus the low altitude group.

*^#^P < 0.05 versus the H1 group.*

### Pharmacokinetics

**FIGURE 3:**
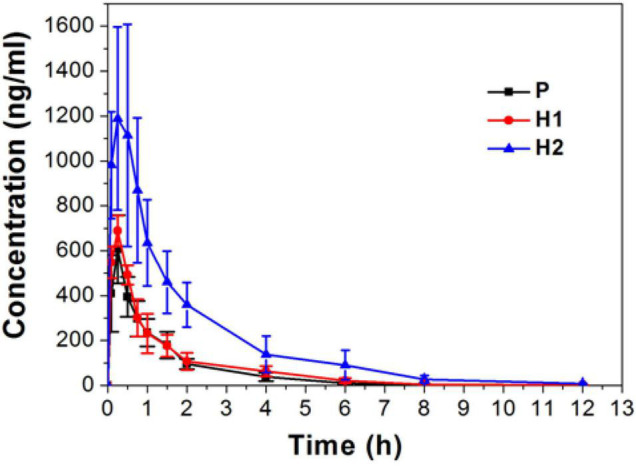
Plasma concentration-time profiles of sildenafil in rats at low altitude and after acute exposure to high altitude.

This study found significant alterations in the pharmacokinetics of sildenafil under the special environment of high altitude hypoxia. The mean pharmacokinetic parameters of sildenafil are listed in Table 2. The concentration-time profiles in plasma obtained from the three groups have similar shapes. Figure 3 shows mean plasma concentration-time profiles for sildenafil. There were no statistically significant differences in pharmacokinetic parameters between the group P and group H1. Compared with the P group, the area under the curve-time curve (AUC) and the mean residence time (MRT) in the H2 group increased significantly and increased by 213.32 and 72.34% in the 0–24 h. The half-life (t_1/2_) and peak concentration (C_max_) increased by 44.27 and 133.67%, respectively. Plasma clearance (CL) and apparent volume (V) decreased by 69.13 and 46.75%, respectively. Compared with the H1 group, the AUC and MRT of the H2 group increased significantly and increased by 160.02 and 30.12% within 0–24 h. The t_1/2_ and C_max_ increased by 47.69 and 105.54%, respectively. The clearance and volume distribution values decreased by 61.85 and 44.55%, respectively. It can be seen from the parameter changes that the process of absorption, distribution, metabolism and excretion of sildenafil in rats is changed after acute hypoxia.

**TABLE 2:** The main pharmacokinetic parameters of sildenafil in rats at low altitude and after acute exposure to high altitude (Mean ± SD, *n* = 8).

Parameters	Low altitude (P)	Acute exposure (H1)	Acute exposure (H2)
AUC_0–24/_μg⋅L^–1^⋅h^–1^	747.96 ± 78.34	901.26 ± 65.78	2343.43 ± 131.20*^#^
AUC_0–8/_μg⋅L^–1^⋅h^–1^	751.81 ± 76.90	906.58 ± 66.31	2356.57 ± 131.29*^#^
MRT_0–24/_h	1.41 ± 0.18	1.66 ± 0.32	2.16 ± 0.74*^#^
MRT_0–8/_h	1.47 ± 0.21	1.72 ± 0.34	2.25 ± 0.77*^#^
t_1/2_/h	1.07 ± 0.22	1.30 ± 0.39	1.92 ± 0.73*^#^
T_max_/h	0.25 ± 0.00	0.25 ± 0.00	0.28 ± 0.19
CL/L⋅h^–1^	83.52 ± 11.86	67.57 ± 10.07	25.78 ± 4.22*^#^
Vd/L	133.07 ± 27.19	127.80 ± 24.37	70.86 ± 18.13*^#^
C_max/_μg⋅L^–1^	605.83 ± 41.85	688.75 ± 57.31	1415.67 ± 84.40*^#^

*P < 0.05 versus the low altitude group.

*^#^P < 0.05 versus the H1 group.*

### Effect of Hypoxia on the Biochemical Indicators

The results of the biochemical analysis are listed in Table 3. Compared with the low altitude group, it shows that the AST values of the acute exposure group (H1) were significantly increased. The TP, ALB, AST, ALT, CRP, BUN, and UA levels of the high altitude group (H2) were significantly increased by 13.72 ± 1.61, 12.71 ± 1.29, 28.18 ± 5.15, 54.81 ± 5.15, 18.53 ± 0.83, 13.11 ± 4.09, and 16.45 ± 0.80%, respectively, compared with those in the plain group (*p* < 0.05), but the Cr levels was severely reduced by 30.97 ± 8.18% (*p* < 0.05). The TP, ALB, AST, ALT, CRP, BUN, and UA levels of the H2 group increased by 10.31 ± 1.34, 14.86 ± 0.55, 15.78 ± 8.25, 59.64 ± 7.75, 18.34 ± 3.94, 27.19 ± 1.18, and 23.81 ± 5.23%, respectively, compared with the H1 group, whereas the Cr levels significantly decreased.

**TABLE 3:** The comparison of main biochemical parameters between the three groups (mean ± SD, *n* = 8).

Biochemical parameters	Low altitude (P)	Acute exposure (H1)	Acute exposure (H2)
ALP (IU/L)	106.75 ± 13.29	101.50 ± 23.34	110.38 ± 26.22
TP (g/L)	52.13 ± 1.66	53.74 ± 1.78	59.28 ± 2.50*^#^
ALB (g/L)	22.43 ± 0.75	22.01 ± 0.92	25.28 ± 1.04*^#^
AST (IU/L)	79.38 ± 5.26	87.88 ± 16.68*	101.75 ± 9.35*^#^
ALT (IU/L)	33.75 ± 3.24	32.73 ± 5.97	52.25 ± 31.32*^#^
TBIL (μmol/L)	7.84 ± 0.70	8.20 ± 0.63	8.30 ± 0.19
DBIL (μmol/L)	−0.68 ± 0.12	−0.79 ± 0.31	−1.25 ± 0.73*^#^
CRP (mg/mL)	12.41 ± 1.05	12.43 ± 1.53	14.71 ± 2.02*^#^
TCHO (mmol/L)	1.19 ± 0.05	1.18 ± 0.15	1.39 ± 0.31
BUN (mmol/L)	4.88 ± 0.39	4.34 ± 0.71	5.52 ± 1.22*^#^
Cr (μmol/L)	20.30 ± 3.89	21.38 ± 3.44	15.50 ± 2.23*^#^
UA (μmol/L)	154.19 ± 8.83	145.03 ± 11.7	179.56 ± 21.10*^#^

*P < 0.05 versus the low altitude group.

*^#^P < 0.05 versus the H1 group.*

### Comparison of Pathological Changes

**FIGURE 4:**
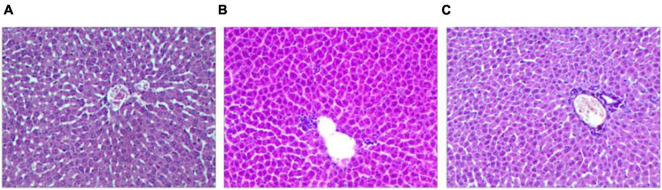
Hematoxylin and eosin staining of rat liver tissue (×200). **(A)** The plain group(P). **(B)** The high altitude group (H1). **(C)** The high altitude group (H2).

The results show significantly pathological changes in the liver among the groups. The liver lobules of group P were integrity and clarity, the liver cells were arranged neatly (Figure 4A). Compared with group P, the rats in group H1 and group H2 revealed the liver injury and inflammation, had much inflammatory cell infiltration and edema (Figures 4B,C).

### Effects of High Altitude on CYP3A4 Protein Expression

**FIGURE 5:**
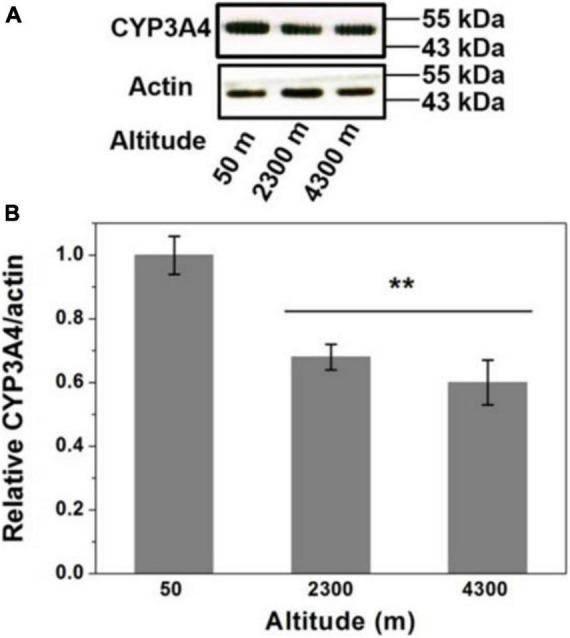
Western blots analysis of protein expression of CYP3A4 in the liver. **(A)** Downregulation of protein expression of CYP3A4 in high altitude. Actin was used as a loading control. **(B)** The quantification of the blots. The data are expressed as the means ± SE of three experiments. **P < 0.01 versus the low altitude group (50 m).

Protein expression levels of CYP3A4 in the liver were studied using western blot (Figure 5). The results showed a remarkable decrease in the level of CYP3A4 protein in 2300 and 4300 m groups. High altitude could significantly down-regulate the protein expression of CYP3A4 compared to the plain group. The values for group 4300 m were significantly decreased by 40% compared with group 50 m (*p* < 0.05).

## Discussion

First, the blood gas index of each group of rats was measured, because its changes can prove the hypoxia of the body and affect the pharmacokinetic characteristics of the drug (Wang et al., 2016). Previous studies have shown that hypoxia increases hemoglobin levels in rats and cells (Grek et al., 2011; Ivy et al., 2021). This study also found that acute hypoxia reduced SaO_2_, PaCO_2_, PaO_2_, and SBC, and increased Lac and Hb. It indicates that the body has symptoms of compensatory respiratory alkalosis and hypoxemia (Palmer, 2012; Brinkman and Sharma, 2021), and it is proved that the rats are in an anoxic state. Blood gas analysis also showed that changes in Na^+^ concentration in the H2 group may affect the absorption function of the gastrointestinal tract, which may further affect drug absorption. Subsequent studies examined the pharmacokinetic characteristics of sildenafil. Before the evaluation of pharmacokinetics, an LC-MS/MS method for the determination of sildenafil was established, which is superior to previous analytical methods in terms of analysis time and accuracy. Pharmacokinetic experiments show that acute high altitude hypoxia does affect the pharmacokinetic characteristics of sildenafil: including absorption, distribution, metabolism, and excretion, and the degree of influence is related to the altitude. The change of Na^+^ concentration in the blood gas index affects the AUC and Cmax of the drug, which mainly affects the absorption process.

Secondly, we analyzed the biochemical indicators of rats because it is a method to assess changes in blood sugar, proteins, enzymes, and metabolites in the body. At the same time, it is an important indicator to evaluate liver and kidney function and drug clearance rate (Li et al., 2015). The results of this experiment showed significant changes in biochemical indicators, especially the decrease in renal clearance. This is one of the important reasons for the decreased clearance of sildenafil in rats after acute hypoxia, *i.e*., changes in biochemical indicators mainly affect the distribution, clearance, and excretion of sildenafil. Several studies have shown that high-altitude environments have varying degrees of impact on human physiological functions and their circadian rhythms, brain functions, and behavioral functions (Ma, 1988; Rostrup et al., 2005; Xie et al., 2020; Wei et al., 2021). Therefore, it is expected that similar results may be observed in humans in this study. The pharmacokinetic study of sildenafil in high-altitude populations is currently underway. In addition, it should be pointed out that this study alone cannot fully recommend clinical dosages. Thus, health care providers can consider recommending that patients with acute hypoxia should pay attention to medication and seek medical attention if they have adverse reactions.

Finally, this study analyzed the pathological changes of rat liver tissue and the expression of CYP3A4. After acute hypoxia, rat liver tissue has many inflammatory cell infiltration and edema, which may affect the metabolism of the drug (Wang et al., 2017). The analysis of CYP3A4 protein expression further indicated that acute hypoxia significantly down-regulated the protein expression of CYP3A4. The half-life of the substrate sildenafil is prolonged, and the average residence time in the body is prolonged, indicating that its metabolism is slowed down. Thus, down-regulation of CYP3A4 protein expression is closely related to the slowing down of sildenafil metabolism (De Denus et al., 2018).

Sildenafil has antioxidant and anti-fatigue properties (Uthayathas et al., 2007; Maziero Alves et al., 2021), it thus may be suitable for people who exercise at altitude. Due to the increasing popularity of sildenafil at high altitude areas, it is important to understand the effects of location altitude on the body and the drug pharmacokinetics. This study demonstrates that most of the pharmacokinetic parameters of sildenafil have changed under acute hypoxic conditions, and there are many influencing factors, and there is a certain correlation between these factors. For example, physiological and pathological changes in the body and changes in metabolic enzymes are important factors influencing the changes in the pharmacokinetic parameters of sildenafil. The concentration of the drug in the body must be maintained within the therapeutic window to play its due role. Although there are reports that food reduces the rate and extent of systemic exposure to sildenafil, these reductions are not clinically meaningful (Nichols et al., 2002). In addition, compared with humans, the apparent volume of distribution of sildenafil in rats has no significant change, but the clearance rate is increased and the elimination half-life is shortened (Nichols et al., 2002). Thus, our current results are necessary for reference when conducting subsequent human trials. In aggregate, this study provides a valuable reference and new ideas for the study of drug metabolism under acute hypoxic conditions and guides clinical rational drug use and personalized drug use in high altitude areas.

## Conclusion

In conclusion, we demonstrated that the acute hypoxia down-regulates the protein expression of the metabolic enzyme CYP3A4, and at the same time demonstrates that there is a multi-factor regulation mechanism in the changes of the substrate sildenafil pharmacokinetic process, which is closely related to the changes of blood gas, biochemical indicators and metabolic enzymes.

## Data Availability Statement

The original contributions presented in the study are included in the article/supplementary material, further inquiries can be directed to the corresponding author.

## Ethics Statement

The animal study was reviewed and approved by 940th Hospital of Joint Logistics Support Force of CPLA.

## Author Contributions

JZ and RW designed the research, analyzed the results, and performed most laboratory experiments. JZ drafted and revised the manuscript, analyzed the results, drafted the figure, performed part laboratory experiments and assisted all laboratory experiments. JZ and RW. Both authors contributed to the article and approved the submitted version.

## Conflict of Interest

The authors declare that the research was conducted in the absence of any commercial or financial relationships that could be construed as a potential conflict of interest.

## Publisher’s Note

All claims expressed in this article are solely those of the authors and do not necessarily represent those of their affiliated organizations, or those of the publisher, the editors and the reviewers. Any product that may be evaluated in this article, or claim that may be made by its manufacturer, is not guaranteed or endorsed by the publisher.
